# Enteric pathogens and cellular transformation: bridging the gaps

**DOI:** 10.18632/oncotarget.2384

**Published:** 2014-08-26

**Authors:** Shahid Umar

**Affiliations:** ^1^ Department of Molecular and Integrative Physiology, University of Kansas Medical Center, Kansas City, KS

**Keywords:** EMT, Slug, Snail, Twist, Zeb1, Wnt, Notch

## Abstract

Cancer patients in general, either due to the nature of their underlying illness or because of being on chemotherapeutic regimens, are at increased risk of infection. Indeed, microbes can exploit the innate plasticity of the epithelial cells to promote their trans-differentiation into a mesenchymal phenotype in a process called epithelial-to-mesenchymal transition (EMT). This process as well as the reverse, mesenchymal-epithelial transition, occurs repeatedly during normal embryonic development and is recapitulated during pathologies such as tissue fibrosis or tumor metastasis. Multiple signaling pathways including TGFβ, Wnt and Notch working together with transcription factors such as Slug, Snail, Twist, Zeb1 and 2 suppress E-cadherin, induce EMT and result in loss of cell-cell adhesion, increased tumor progression and migration. In addition, in approximately 20% of all cases, microbial organisms including pathobionts of the commensal microbiota, have been implicated in inflammatory processes that promote tumor growth. Thus, the dynamic process of EMT serves to enhance tumor progression and is also involved in the generation of cancer stem cells (CSCs) across multiple organ systems including colon cancer. Suffice to say, EMT and CSC molecular pathways activated by pathogens, may represent a unique therapeutic alternative to conventional anti-neoplastic strategy to mitigate early stage metastasis and/or frank malignancy.

Cancer is no longer a problem of cancer cells alone since the development and metastasis of cancers clearly involves many aspects of the host. There is growing evidence for a direct mechanistic relationship between the changes induced by inflammation and epigenetic deregulation during tumor development and progression. The proclivity of tumor cells to undergo widespread dissemination to distant sites is the major cause of cancer mortality. Consequently, this process has been extensively studied, but the exact nature of the events that may contribute to, or are essential for, metastasis remain controversial. During malignant progression, it has been proposed that carcinoma cells undergo an epithelial-to-mesenchymal transition (EMT), in which they lose epithelial characteristics and acquire invasive properties and stem cell-like features (Fig. 1) [1]. Multiple signaling pathways including TGFβ, Wnt and Notch working together with transcription factors such as Slug, Snail, Twist, Zeb1 and 2 suppress E-cadherin and induce EMT resulting in loss of cell-cell adhesion and increased tumor progression and migration [2]. Moreover, gene expression patterns in human cancers indicate that de-differentiated cancer cells combine EMT properties with a stem-cell like phenotype to generate migrating cancer stem cells as the basis of metastasis [3]. Indeed, EMT activators such as Twist1, can co-induce EMT and stemness properties thereby linking EMT to cancer stem cells (CSCs) [4].

**Figure 1:**
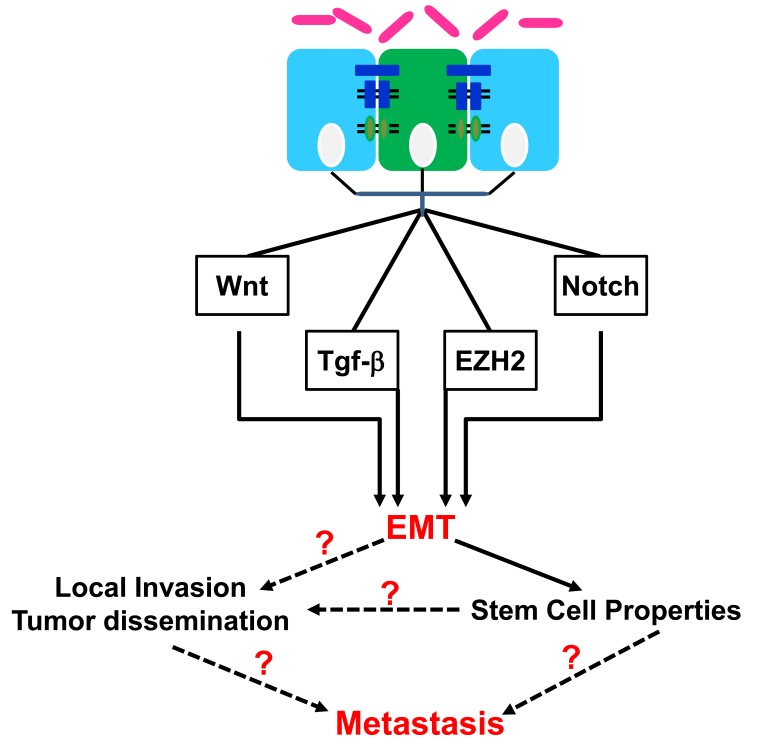
Epithelial-to-mesenchymal transition (EMT) pathways deregulated in cancer and the downstream effects *Citrobacter rodentium* (CR) binding to the luminal surface of the large intestine in a susceptible host can influence the epigenetic signaling within the stem (green) and/or progenitor (blue) cells to promote inflammation and EMT. The diagram demonstrates a variety of effector pathways for EMT as well as the downstream consequences related to either local invasion or distant metastasis. Dotted lines and question marks represent the current focus in our laboratory.

Remarkably, several of the EMT-signaling pathways such as TGFβ, Wnt, Notch and NF-κB are also induced by microbial pathogens, therefore suggesting that pathogens may also be considered as EMT inducers [2]. It is noteworthy that bacterial or viral infections, and the resulting chronic inflammation, have been shown to predispose individuals to certain types of cancer. Recent studies also provide evidence of histone modification and chromatin remodeling as key regulators of eukaryotic transcription and therefore excellent targets for pathogenic infection [5]. Epigenetic remodeling including DNA methylation and posttranslational histone modifications, have recently been implicated in the regulation of EMT and tumor cell invasion [6]. Epigenetic dysregulation may contribute to inflammation-driven diseases including cancer, and can lead to the inappropriate silencing of genes necessary to inhibit cancer development. Growing evidence suggests a strong link of enhancer of zest homolog-2 (EZH2), a polycomb repressor complex-2 protein, to oncogenesis and to cancer-specific gene silencing. Overexpression of EZH2 has been found in many cancers and the expression level is correlated with tumor progression and poor prognosis [7].

Surprisingly, much of what we have learned about the process of EMT has come from elegant studies in tumor cell lines [8], while the characterization of the pathways leading to EMT *in vivo* is less clear. Rhim AD et al. [9] by doing lineage tracing in a pancreatic cancer model, recently demonstrated that cells acquire an invasive and metastatic phenotype before detectable tumor formation. Similarly, a recent study demonstrated expression of EMT markers during the pathogenesis of fistulae in Crohn's disease [10]. Utilizing an *in vivo* model of *Citrobacter rodentium* (CR)-induced pre-neoplastic colonic crypt hyperplasia, we have recently shown that hyperplastic crypts when cultured *in vitro*, exhibit EMT-like changes that promotes tumorigenesis following a second hit [11]. We have also discovered that Dclk1 and Lgr5 as markers of quiescent and rapidly cycling stem cells [12], are differentially expressed during progression and regression phases of crypt hyperplasia [11]. In addition, the process of EMT was epigenetically regulated as CR-induced upregulation of EZH2 levels at peak crypt hyperplasia was associated with E-cadherin promoter leading to its downregulation which is the hallmark of EMT process. Thus, it is not unprecedented for either the hyperplastic crypts or intestinal adenomas such as those seen in mice mutant for *Apc* gene [13], to serve as precursors of malignant tumors before detectable local invasion or may be even metastasis. Interestingly, the CR-induced EMT was almost completely attenuated following blockade of the Wnt/β-catenin and Notch cross-talk *in vivo* [11]. Since these pathways are also implicated in epithelial stem cell/CSCs maintenance and proliferation and are generally thought as the cells of origin for a large proportion of CRCs, our studies provide an excellent platform to link enteric pathogens to the cancers of the large intestine and how they can promote either the onset or propagation of EMT signal that promotes metastasis and frank malignancy. Moreover, utilization of CR infection that models human EPEC and EHEC, to study pathogenesis of EZH2-mediated changes in Wnt signaling is clinically relevant and can lead to exciting data to treat diseases with infectious etiology.

Microorganisms are causally implicated in slightly over 20% of all human cancers. Viruses, bacteria and parasites are among the most important pathogens associated with carcinogenesis. It is therefore imperative to focus on understanding the mechanistic basis of malignant transformation initiated by pathogens, an area that promises exciting prophylactic, diagnostic, and therapeutic applications. In conclusion, even though we still lack novel approaches to overcome therapeutic resistance, a combined regimen of chemotherapy and radiation therapy remains the central strategy to fight cancer. Results of the current study [11] suggest that the acquisition of EMT phenotype triggered by an enteric pathogen could be associated with an early onset metastatic spread in genetically predisposed individuals. Since pathogens associated with either chronic pathologies or those that interfere with innate immunity have been described to induce EMT, targeting EMT and CSC molecular pathways activated by pathogens, may represent a unique therapeutic alternative to conventional anti-neoplastic strategy to mitigate early stage metastasis and/or frank malignancy.
